# Supplementary material for: Single-cell transcriptomics unveil profiles and interplay of immune subsets in rare autoimmune childhood Sjögren’s disease
Source: Commun Biol. 2024 Apr 19;7:481. doi: 10.1038/s42003-024-06124-6 (PMC11031574; doi:10.1038/s42003-024-06124-6)
Supplement: Supplementary file 2 — Supplementary Information [file 42003_2024_6124_MOESM2_ESM.pdf]

Supplementary Table 1. Participant groups compared in this study.

	HC	cSjD		Bx	BxRP	Non-cSjD
		RP-	RP+			
2016 criteria	-	+	+	-	-	-
Bx	-	+	+	+	+	-
RP	-	-	+	-	+	-
Symptoms	X	O	O	O	O	O

Abbreviations: HC, Healthy Control; cSjD, childhood Sjögren's disease; RP, recurrent parotitis; Bx, Biopsy; BxRP, Biopsy positivity with recurrent parotitis; Non-cSjD, non-childhood Sjögren's disease; 2016 criteria, The 2016 ACR/EULAR criteria for SjD.

Supplementary Table 2. List of antibodies and relevant reagents used in this study.

Antibodies or Reagents	Clone	Fluorescence	Source	Cat #	Dilution or Concentration
CD45	HI30	FITC	BioLegend	304038	1:100
CD45	HI30	PE	BioLegend	304039	1:100
CD45	HI30	BV421	BioLegend	304032	1:100
CD45	2D1	SPNIR685	BioLegend	368552	1:100
CD3	HIT3a	FITC	BioLegend	300306	1:100
CD3	SK7	PE-Fire 700	BioLegend	344864	1:100
CD4	OKT4	PerCP-Cy5.5	BioLegend	317428	1:100
CD4	RPA-T4	BV605	BioLegend	300556	1:100
CD4	SK3	PE-Cy5	BioLegend	344654	1:100

CD8	SK1	SB550	BioLegend	344760	1:100
CD25	M-A251	PE-Cy7	BioLegend	356108	1:100
CD127	A019D5	APC	BioLegend	351316	1:100
CD127	A019D5	BV510	BioLegend	351334	1:100
CD152	BNI3	APC	BioLegend	369612	1:100
FOXP3	259D	Pacific Blue	BioLegend	320216	1:50
FOXP3	206D	Pacific Blue	BioLegend	320116	1:50
CD11b	M1/70	PE-Dazzle 594	BioLegend	101255	1:100
CD14	63D3	BV510	BioLegend	367124	1:100
CD14	HCD14	APC-Cy7	BioLegend	325620	1:100
CD16	3G8	BV785	BioLegend	302046	1:100
CD39	A1	PE-Cy7	BioLegend	328212	1:100
TIGIT	MBSA43	EF710	Invitrogen	46-9500-42	1:100
Phospho STAT1	KIKSI0803	EF660	Invitrogen	50-9008-42	1:50
TotalSeq™-B0251 Hashtag 1	LNH-94	NA	BioLegend	394631	1:100
TotalSeq™-B0252 Hashtag 2	LNH-94	NA	BioLegend	394633	1:100
TotalSeq™-B0253 Hashtag 3	LNH-94	NA	BioLegend	394635	1:100
TotalSeq™-B0254 Hashtag 4	LNH-94	NA	BioLegend	394637	1:100
Fc receptor blocker	NA	NA	Miltenyi Biotec	130-059-901	1:100

Fixable viability dye	NA	eFluor 780	eBioscience	65-0865-14	1:8,000
Mouse Anti-Human CD3	OKT3	NA	BD Pharmingen	567107	1.5 μg/ml
CFSE	NA	NA	Invitrogen	C34554	2.5 μM
IFN-γ	NA	NA	R & D system	285-F-100	10 ng/ml
Protease/phosphatase inhibitor	NA	NA	Sigma	PPC1010	1:100

Supplementary Figures and Figure Legend

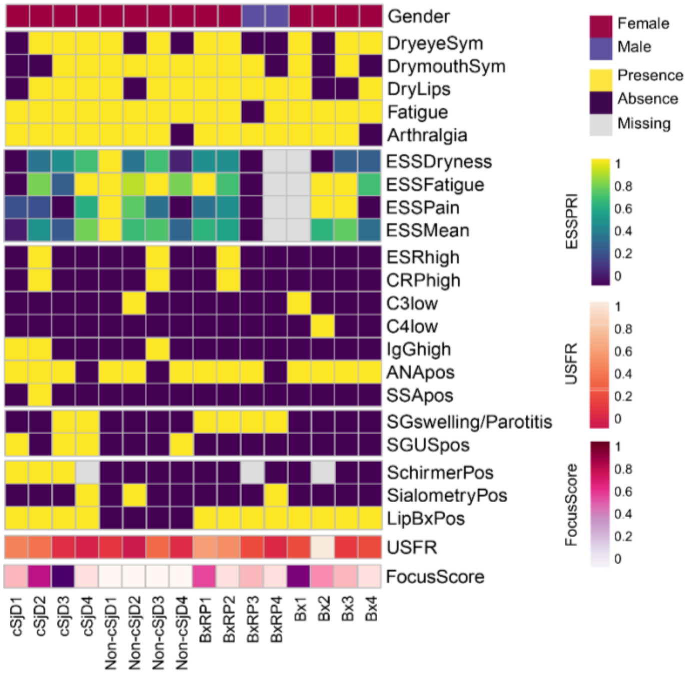

Supplementary Figure 1. Demographic, clinical, and laboratory characteristics of the UF cohort

Clinical features, laboratory parameters, diagnostic tests, medical history, and EULAR Sjögren's syndrome patient reported index (ESSPRI) are presented on the heatmap. Abbreviations: HC, healthy control; cSjD, childhood Sjögren’s disease; Bx, biopsy-positive non-cSjD without recurrent parotitis; BxRP, biopsy-positive non-cSjD with recurrent parotitis; DryeyeSym, reported symptom of dry eyes; DrymouthSym, reported symptom of dry mouth; ESSDryness, ESSPRI dryness domian; ESSFatigue, ESSPRI fatigue domain; ESSPain, ESSPRI pain domain; ESSMean, a mean value of the ESSPRI scores; ESRhigh, high erythrocyte sedimentation rate; CRPhigh, high serum level of C-reactive protein; C3low, low serum level of complement C3; C4low, low serum level of complement C4; IgGhigh, hypergammaglobulinemia; ANApos, positive antinuclear antibody; SSApos, positive anti-Ro/SSA autoantibody; SSBpos, positive anti-La/SSB autoantibody; SGswelling, salivary gland swelling; SGUSpos, positive salivary gland

ultrasonography; SchirmerPos, positive Schirmer's test for dry eyes; SialometryPos, positive sialometry for dry mouth; LipBxPos, positive labial gland biopsy; USFR, unstimulated salivary flow rate; FocusScore, the number of mononuclear cell infiltrates containing at least 50 inflammatory cells in a 4 mm^2 glandular section. The focus score is presented as a normalized value on the heatmap.

Supplementary Figure 2. Pre-processing of the scRNA-seq data in Seurat

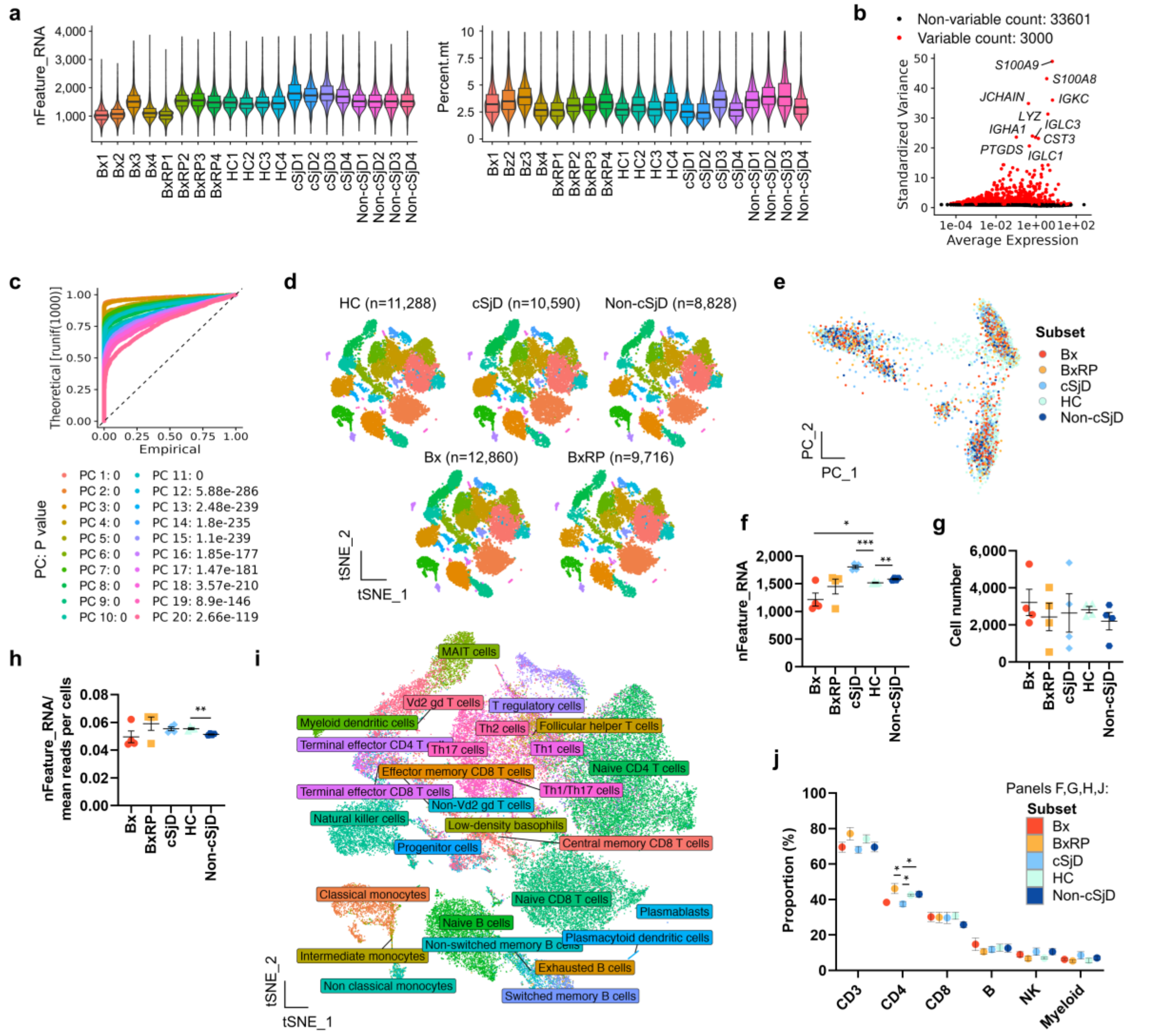

Supplementary Figure 2. Pre-processing of the scRNA-seq data in Seurat

a Selection and filtration of cells based on the number of unique genes detected in each cell (nFeature_RNA) and percentage of reads that map to the mitochondrial genome (Percent.mt). Cells that have unique feature counts over 200 and less than 3,000 and < 10% mitochondrial counts are present on the violin plot. Line in the box plot indicates median value between 25% and 75% quantile. **b** Identification of highly variable features in the scRNA-seq datasets. A total of 3,000 features that exhibit high cell-to-cell variation in the integrated scRNA-seq dataset are selected.

Representative top 10 highly variable features are shown. **c** Principal components showing strong enrichment of features with low p-values are visualized by the JackStraw plot. Immune cell tSNE for each group with cell numbers (**d**) and PCA (**e**) distribution by the group. Overall, there is no difference in the tSNE or PCA distribution of immune cells across the groups. **f** The average value for the unique genes detected in a single cell from each individual. Error bar refers to standard deviation. **g** Cell numbers detected in the cohort. The average cell numbers among the groups are not significantly different. Error bar refers to standard deviation. **h** nFeature_RNA normalized by mean reads per cell. Error bar refers to standard deviation. **i** Cell type recognition by singleR on the tSNE plot. A reference dataset derived from Monaco Immune Cell Data was used. **j** A proportion (average % of cells) of the major immune subsets presented with a standard deviation for each group. Error bar refers to standard deviation. A comparison between the two groups of interest by a two-tailed unpaired Student's t-test. * $p< 0.05$, ** $p< 0.01$, and *** $p < 0.001$. Abbreviations: PC, Principal components; HC, healthy control; cSjD, childhood Sjögren's disease; BX, biopsy-positive non-cSjD without RP; BXRP, biopsy-positive non-cSjD with RP.

Supplementary Figure 3. Overall study scheme and major immune cell types identified by scRNA-seq

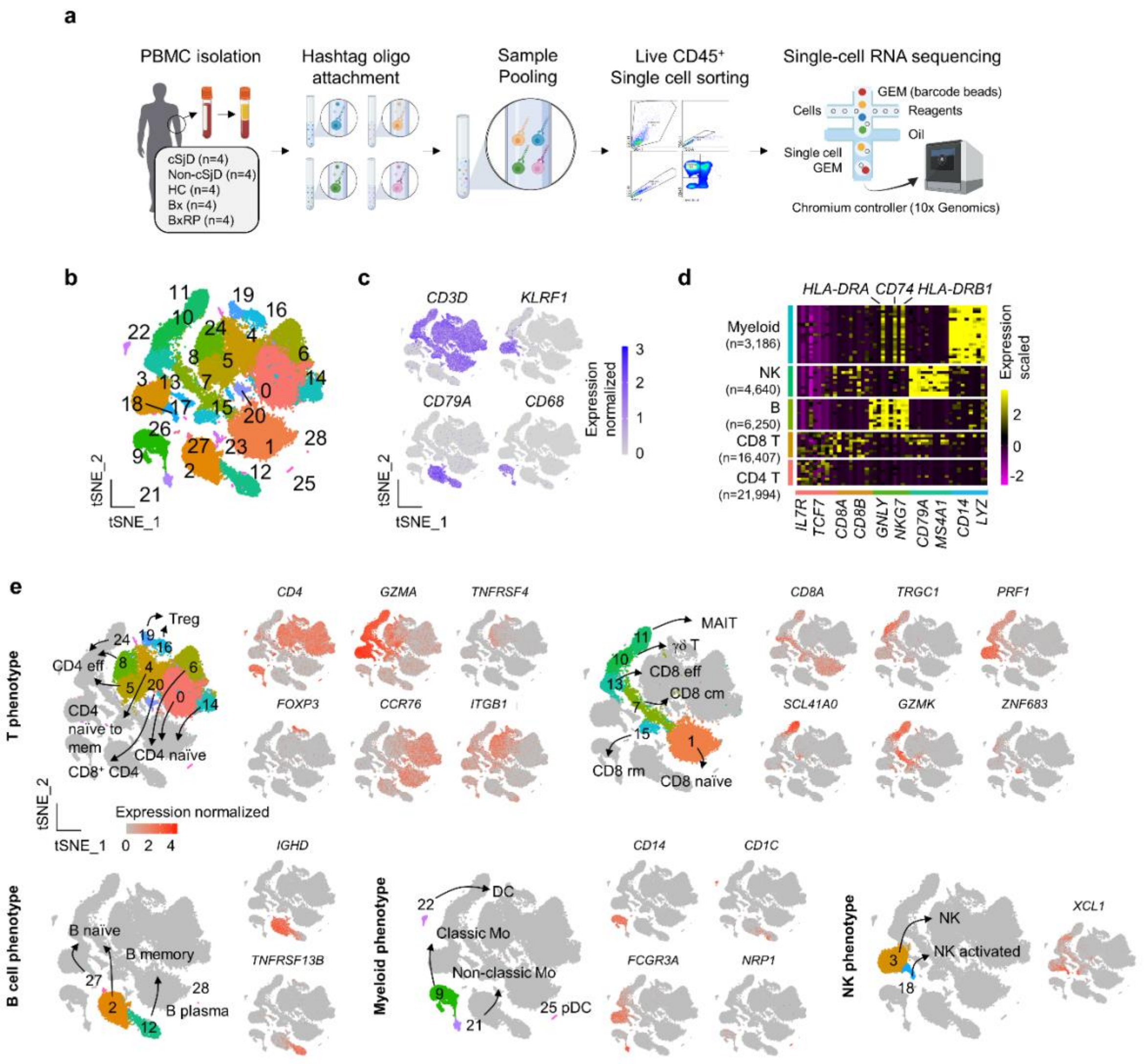

Supplementary Figure 3. Overall study workflow and major immune cell subsets in the PBMC of the UF cohort

a Each biological replicate is hash-tagged with different oligo-conjugated TotalSeq antibodies, pooled, and flow-sorted. Live single peripheral immune cells from cSjD, non-cSjD, Bx, BxRP, and HC (4 samples per group) were subjected to library preparation and bioinformatics analysis. BioRender software was used to create the scientific images under an academic license. **b** A total of 29 clusters identified from the integrated Seurat dataset are presented on the tSNE plot. **c**

Canonical markers for T, B, NK, and myeloid cells are present on the tSNE plot. **d** Representative genes are presented on the heatmap among the top 20 DEGs to define each major immune subset. The total number of the cells analyzed is presented below the immune cell subsets. **e** Representative canonical markers to identify functionally distinct immune subsets are shown. Abbreviations: HC, healthy control; cSjD, childhood Sjögren's disease; BX, biopsy-positive non-cSjD without RP; BXRP, biopsy-positive non-cSjD with RP.

Supplementary Figure 4. Analysis of cSjD myeloid subsets

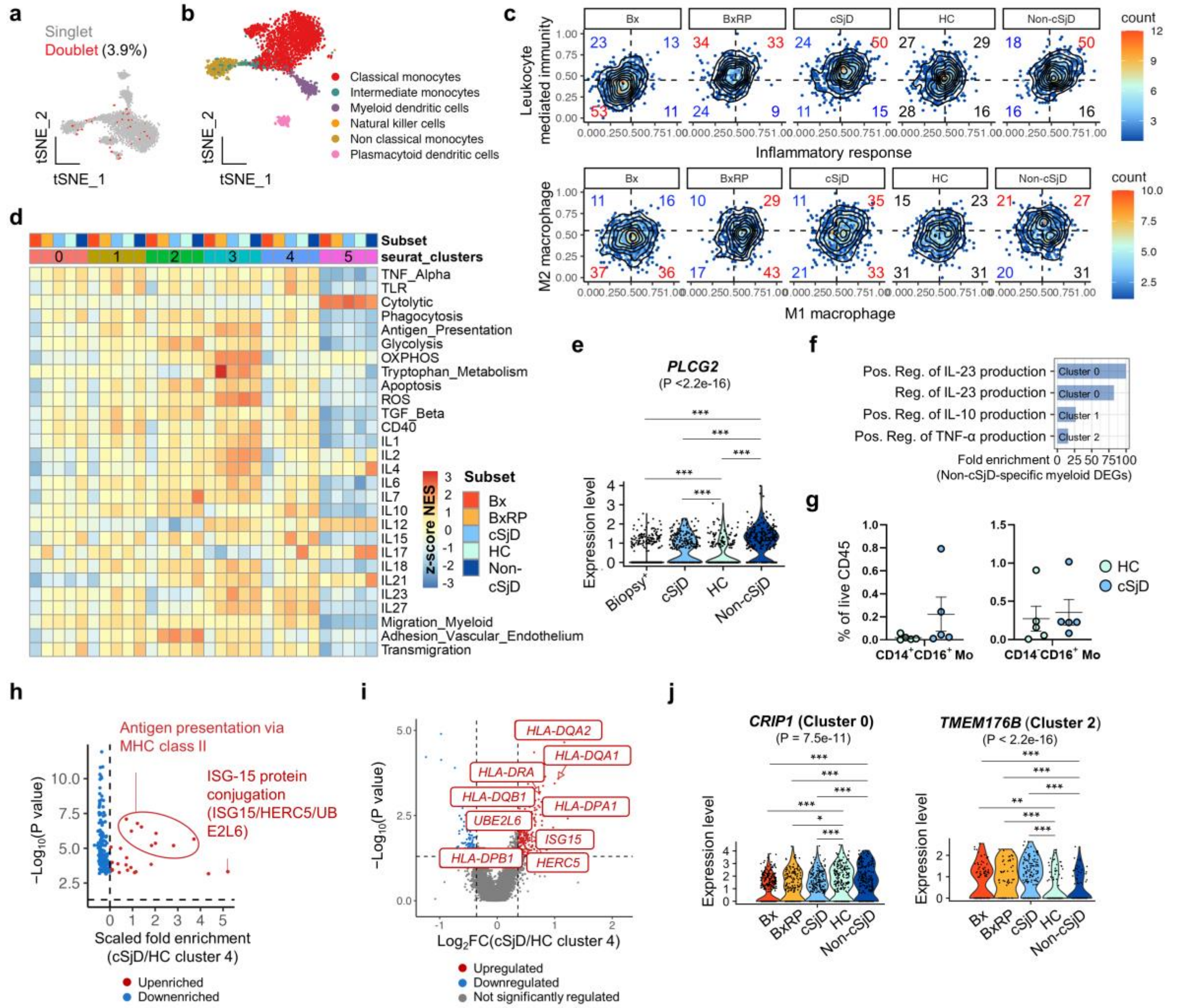

Supplementary Figure 4. Analysis of cSjD myeloid subsets

a Distribution of potential doublets and doublet percentages in the myeloid population. **b** Cell type recognition by singleR on the tSNE plot. A reference dataset derived from Monaco Immune Cell Data is used. **c** GSEA and visualization of results using hex density enrichment plot. Hex density enrichment plot reveals the enrichment pattern of the indicated immune-related pathways in the myeloid population across groups. Myeloid cells in the patient cohort are more inflammatory, compared to HC. Note that non-cSjD patients have heterogeneous myeloid populations polarized with either M1 or M2 macrophage signatures. Figures colored by red and blue numbers on each

quadrant respectively illustrate upward and downward trends, compared to HC. Count refers to the number of cells located in the hexagon. **d** GSEA. Immune-related pathways, such as cytokine, metabolism, and antigen presentation, are present across myeloid subsets and groups. **e** Violin plot reveals a specific expression pattern of *PLCG2* in myeloid cells from non-cSjD patients. P values obtained from significance testing utilizing one-way analysis of variance available within the ggpubr R package are presented. Statistical significance was obtained by comparing two groups of interest with a non-parametric Wilcoxon rank-sum t-test). * $P < 0.05$, ** $P < 0.01$, and *** $P < 0.001$. **f** Gene ontology analysis using non-cSjD-specific myeloid DEGs shows enrichment of biological processes associated with IL-10, IL-23, and TNF-α. **g** Flow cytometric analysis of PBMCs reveals no fractional change in the intermediate or non-classic monocyte between cSjD and HC. Error bar refers to standard deviation. **h** Gene ontology analysis using cSjD DEGs reveals antigen presentation and ISG15 protein conjugation being significantly enriched in cluster 4. Blue and red dot refers to gene ontology term whose scaled fold enrichment is arbitrarily below and above 0, respectively. **i** Genes enriched in the gene sets of (**h**), such as *ISG15*, *HERC5*, and *UBE2L6*, are significantly upregulated in the cluster 4 monocytes in cSjD compared to HC. Dot or gene colored by blue and red refers to down and up regulated DEGs, respectively. **j** Biopsy-related myeloid DEGs are presented in the violin plot. ANOVA available within the ggpubr R package and non-parametric Wilcoxon rank-sum t-test for two group comparisons were performed. * $p < 0.05$, ** $p < 0.01$, and *** $p < 0.001$. Abbreviations: HC, healthy control; cSjD, childhood Sjögren's disease; BX, biopsy-positive non-cSjD without RP; BXRP, biopsy-positive non-cSjD with RP.

Supplementary Figure 5. Analysis of SjD myeloid subsets

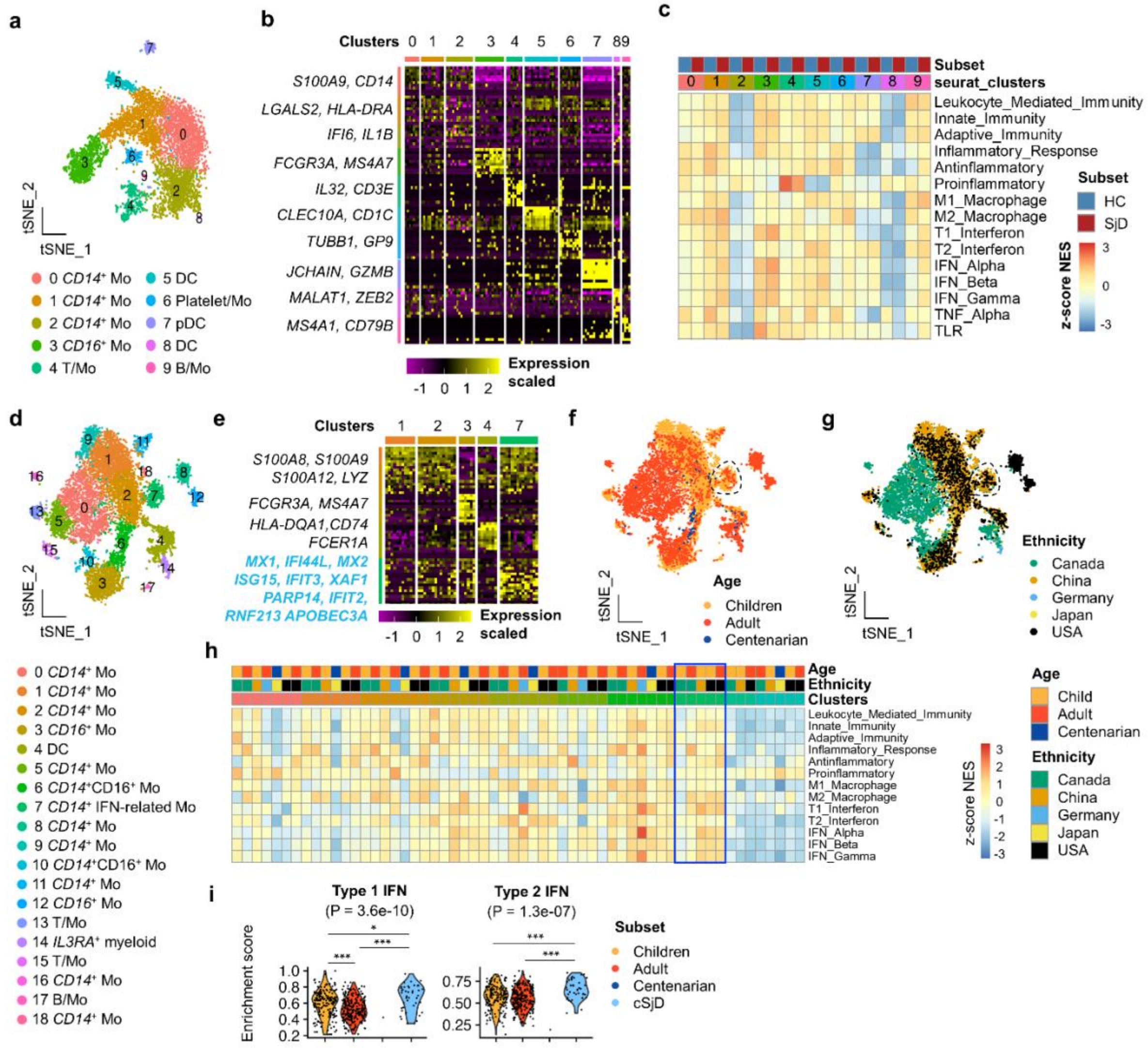

Supplementary Figure 5. Analysis of myeloid subsets from SjD, healthy children, adults, and centenarian

a Analysis of published scRNA-seq data from SjD (GSE157278) to characterize myeloid subpopulations. A total of 7 major myeloid subsets are identified after the sub-clustering of *CD68*$^{+}$ cells. **b** Among the top 20 differentially regulated genes to define each subset, representative genes are presented on the heatmap. Representative genes defining each cluster are present. **c** GSEA reveals that myeloid cells from SjD compared to HC have no marked enrichment pattern with gene

sets associated with inflammation and immunity, including IFN responses. **d** Analysis of published scRNA-seq data to characterize *CD68*$^{+}$ myeloid subpopulations from healthy children (GSE148633, GSE206295, GSE168732, phs003048.v1.p1), adult (GSE148633, GSE216489, GSE211560), and centenarian (hum0229.v1). **e** Among the top 10 differentially regulated genes to define each subset, representative genes and clusters are presented on the heatmap. tSNE distribution by the age (**f**) and ethnicity (**g**). IFN-related monocytes are noted in dash line. **h** GSEA reveals that myeloid cells from children compared to adults have no enrichment pattern with gene sets associated with inflammation, including IFN signatures. **i** Enrichment scores of IFN-related gene signatures in IFN-related monocytes across groups are presented in the violin plot. ANOVA available within the ggpubr R package and non-parametric Wilcoxon rank-sum t-test for two group comparisons were performed. * $p< 0.05$, ** $p< 0.01$, and *** $p< 0.001$. Abbreviations: HC, healthy control; cSjD, childhood Sjögren's disease; SjD, adult Sjögren's disease

Supplementary Figure 6. Analysis of CD4+ T subsets

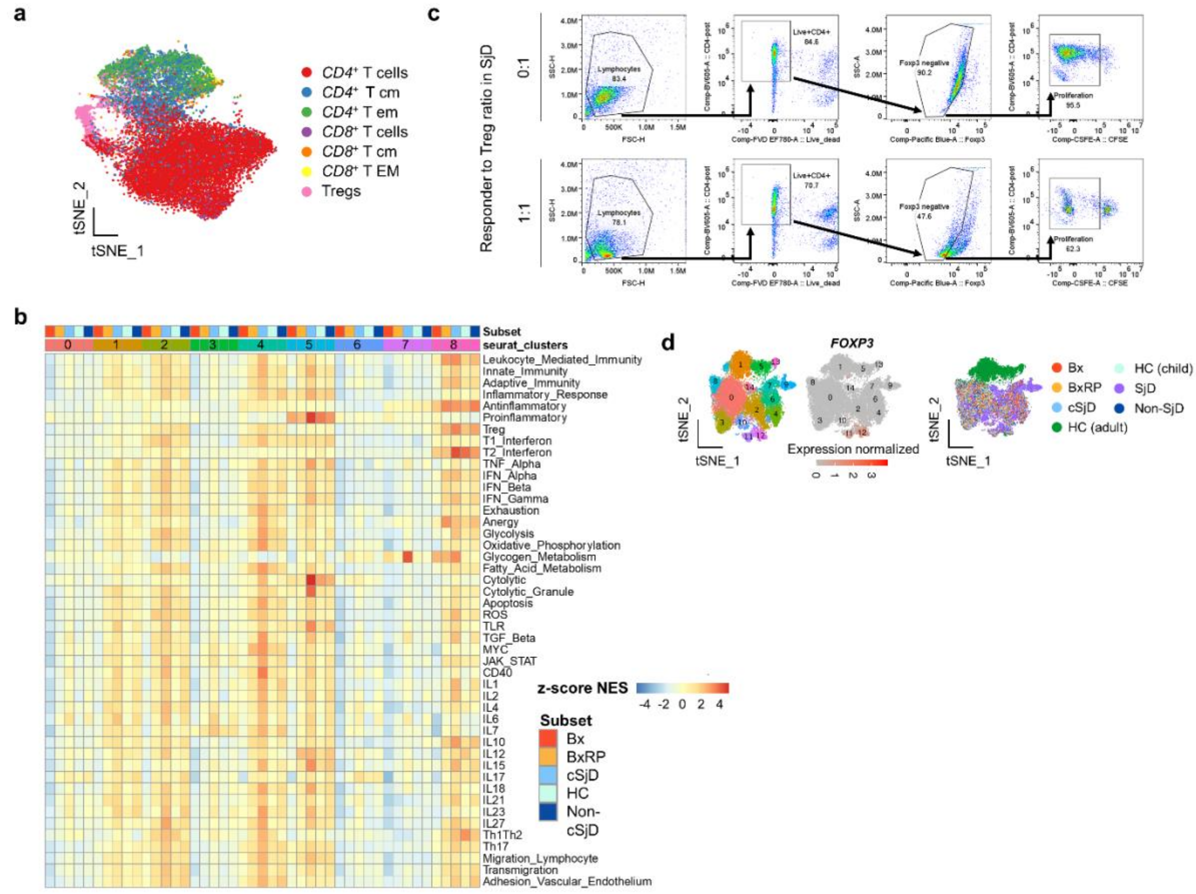

Supplementary Figure 6. Analysis of CD4+ T subsets

a Cell type recognition by singleR on the tSNE plot. A reference dataset derived from Monaco Immune Cell Data was used. **b** All immune-related pathways are present across CD4+ T subsets and the groups. **c** Gaiting strategy to evaluate T cell proliferation while Treg suppression assay related to Figure 3I. Representative flow cytometry panels with antibodies used in SjD samples are present. Live CD4+Foxp3- CFSE-diminishing cells are evaluated for T cell proliferation. **d** Sub-clustering of *CD4+* T cells in cSjD and adult SjD. *FOXP3* gene is used to identify Treg clusters (Clusters 11 and 12). Distribution of CD4+ T subsets is present on the tSNE (right) by the group. Overall, *CD4+* T cells from adult HC are found to be separately distributed on the dimensional plot, suggesting a different global pattern of RNA expression. **e** Compositional changes of *CD4+* T

subsets across the groups. **f** Cell cycle profile across *CD4*$^{+}$ T subsets and groups. Abbreviations: HC, healthy control; cSjD, childhood Sjögren's disease; BX, biopsy-positive non-cSjD without RP; BXRP, biopsy-positive non-cSjD with RP

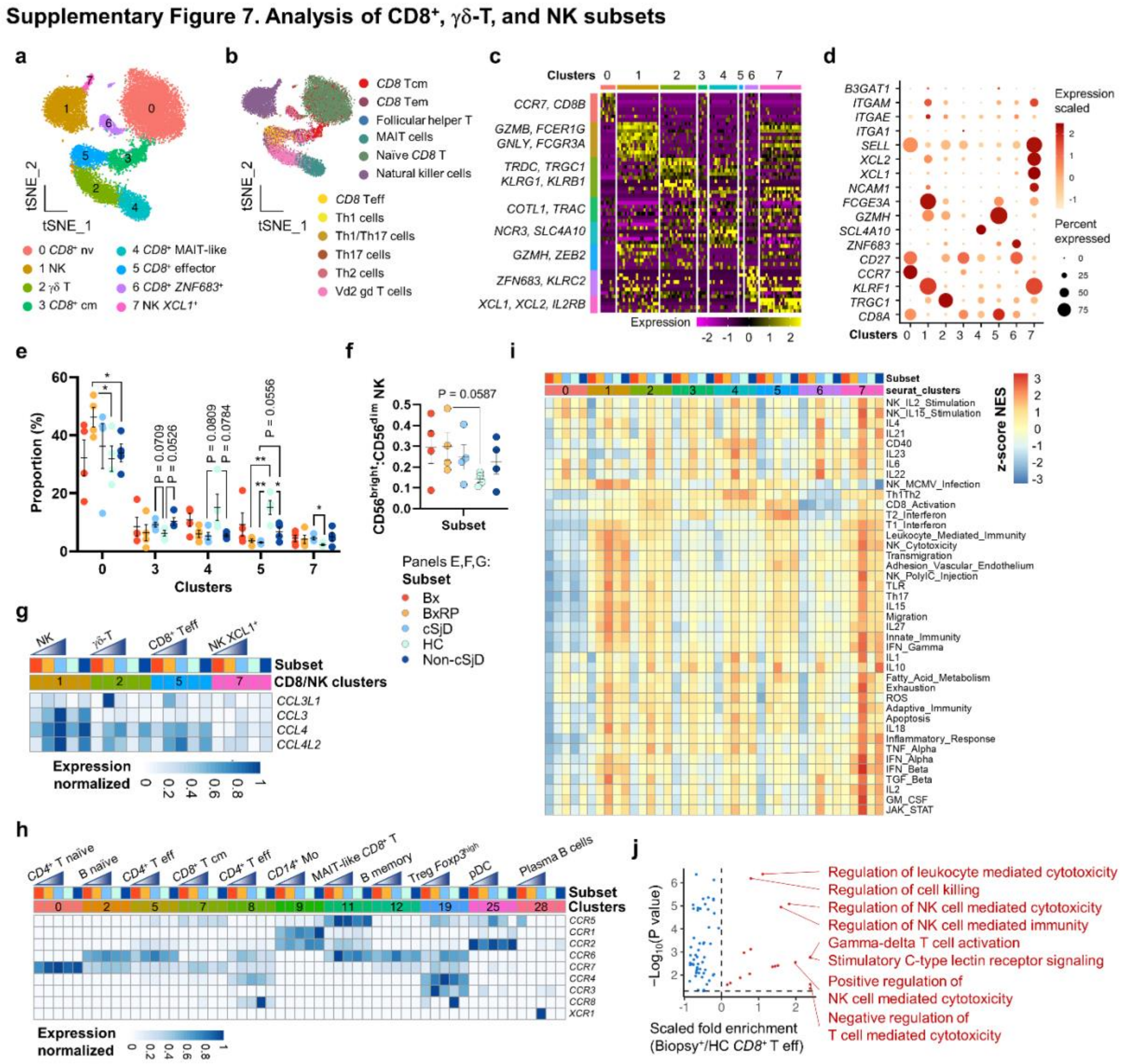

Supplementary Figure 7. Analysis of *CD8*⁺ T, γδ-T, and NK subsets

a A total of 8 subsets are identified and presented on the tSNE plot. **b** Cell type recognition by singleR. A reference dataset derived from Monaco Immune Cell Data available within the celldex package is used. **c** Representative genes are presented on the heatmap among the top 10 DEGs in each subset. **d** Selective markers used to identify functionally unique immune subsets are presented in the dot plot. **e** Compositional change of immune subsets. Error bar refers to standard deviation. **f** The ratio of Cluster 7 to Cluster 0 NK subsets across groups. Note that BxRP patients tend to

increase the ratio of more activated NK subsets compared to conventional subsets. Error bar refers to standard deviation. Expression pattern of CC chemokines **g** and receptors and XCR1 (**h**) across original subsets and groups on the heatmap. **i** GSEA with immune-related pathways is shown. **j** Gene ontology analysis using DEGs defining in effector $CD8^{+}$ T cells from the $Biopsy^{+}$ group. Blue and red dot refers to gene ontology term whose scaled fold enrichment is arbitrarily below and above 0, respectively. * and ** refer to statistical significance (p-value < 0.05 and 0.01, respectively, calculated by a two-tailed unpaired Student's t-test) of the corresponding subset of the two groups. Abbreviations: HC, healthy control; cSjD, childhood Sjögren's disease; BX, biopsy-positive non-cSjD without RP; BXRP, biopsy-positive non-cSjD with RP.

Supplementary Figure 8. Overall signaling patterns of significant pathways

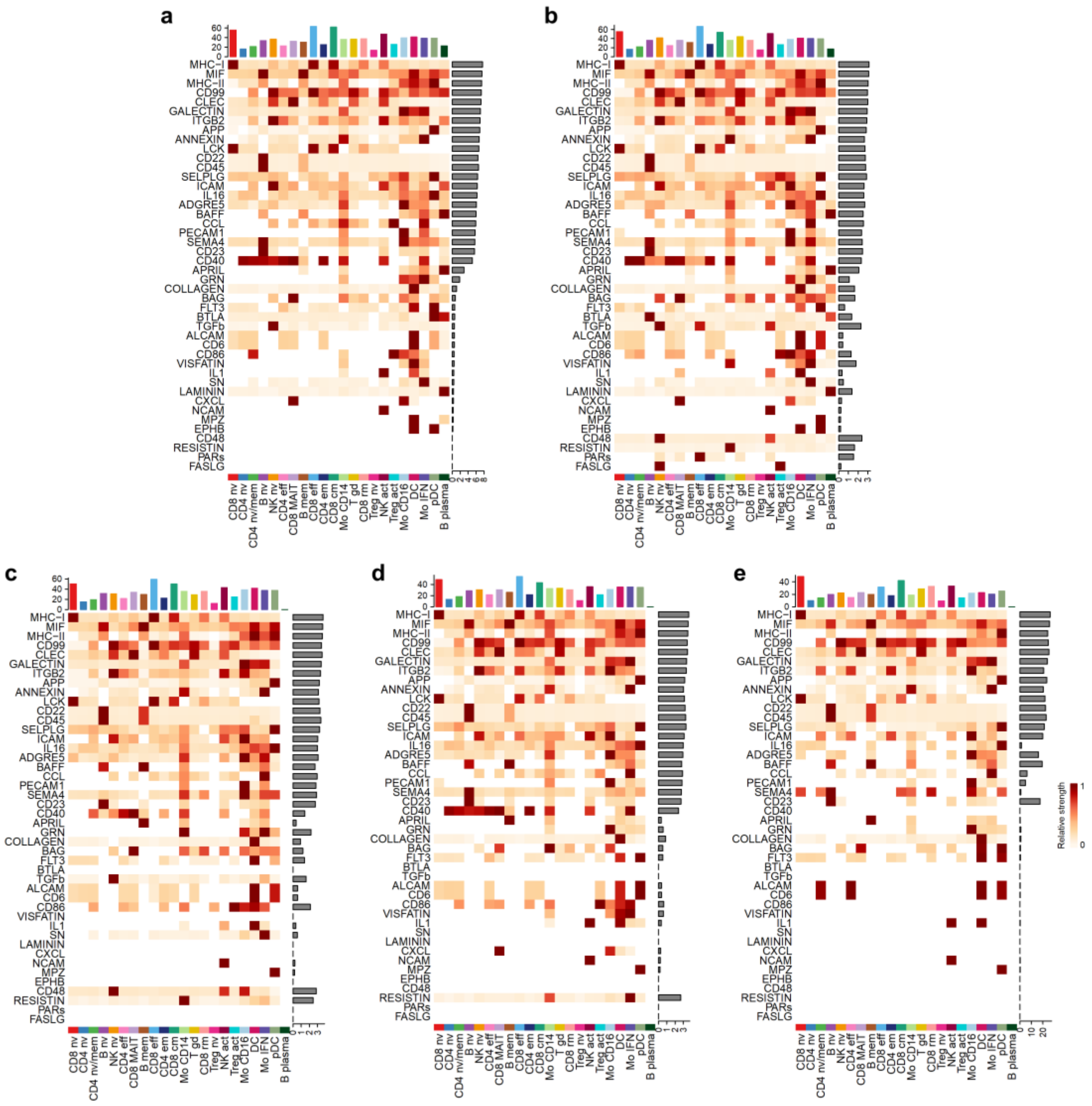

Supplementary Figure 8. Overall signaling patterns of significant pathways

Overall signaling patterns of significant pathways presented in HC control (**a**) and patients with cSjD (**b**) non-cSjD (**c**), BxRP (**d**), and Bx (**e**). Some signaling pathways are only identified in the patient cohort, such as CD48, RESISTIN, PARs, and FASLG. Bars refer to the sum of the original computed interaction strength in each column and row. Relative strength refers to the normalized communication probability sending from one group via one pathway.

Supplementary Figure 9. CellChat-based identification of cell-to-cell interaction specific for non-cSjD

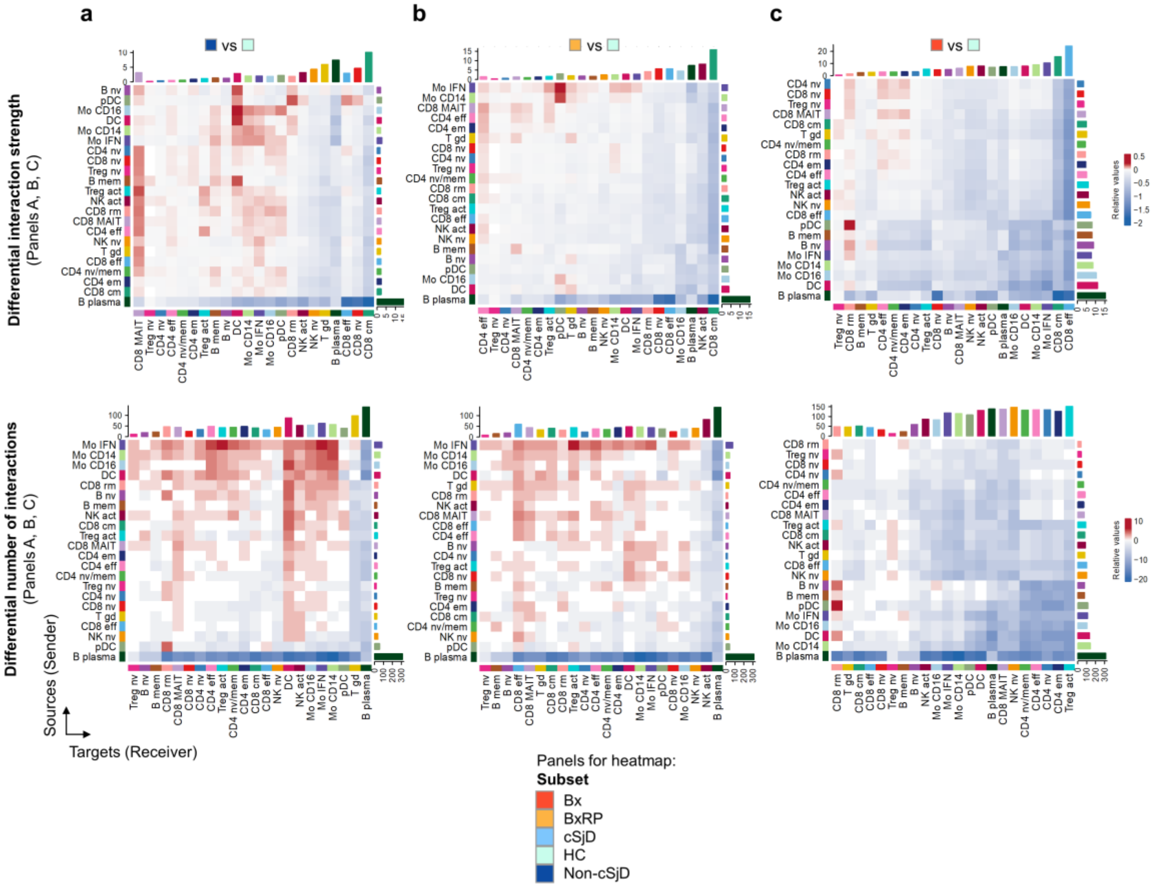

Supplementary Figure 9. CellChat-based identification of cell-to-cell interaction for non-cSjD groups

The overall pattern of differential interaction strength and numbers in (**a**) non-cSjD, (**b**) BxRP, and (**c**) Bx, compared to HC, is shown. Relative values = the interaction strength/number from source to target in comparison group – the interaction strength/number from source to target in control group. Abbreviations: HC, healthy control; cSjD, childhood Sjögren's disease; BX, biopsy-positive non-cSjD without RP; BXRP, biopsy-positive non-cSjD with RP.

Supplementary Figure 10. CellChat-based identification of cell-to-cell interaction specific for SjD

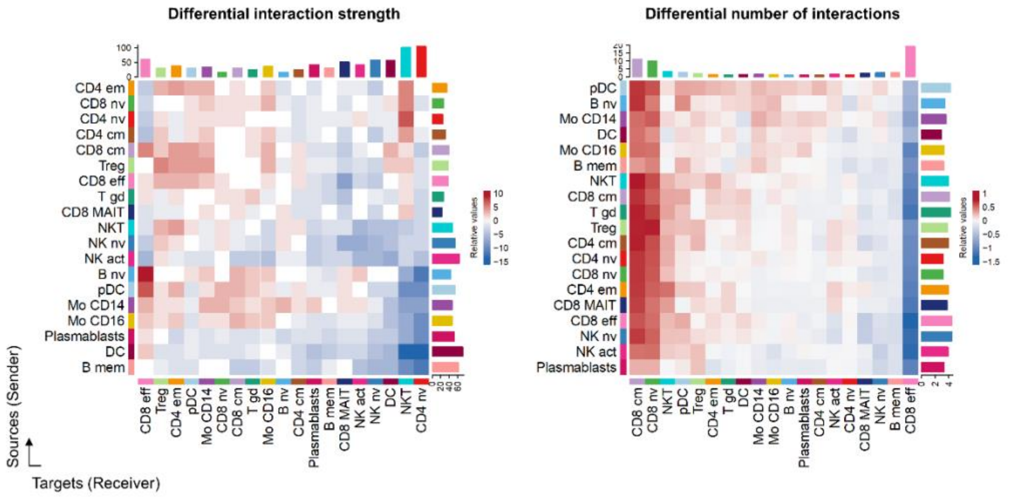

Supplementary Figure 10. CellChat-based identification of cell-to-cell interaction associated with SjD

Analysis of published scRNA-seq data (GSE157278) to characterize cell-to-cell interactions in SjD compared to adult HC. The overall pattern of differential interaction strength across the groups is shown. Relative values = the interaction strength/number from source to target in comparison group – the interaction strength/number from source to target in control group.

Supplementary Figure 11. CellChat-based identification of cell-to-cell interaction specific for cSjD

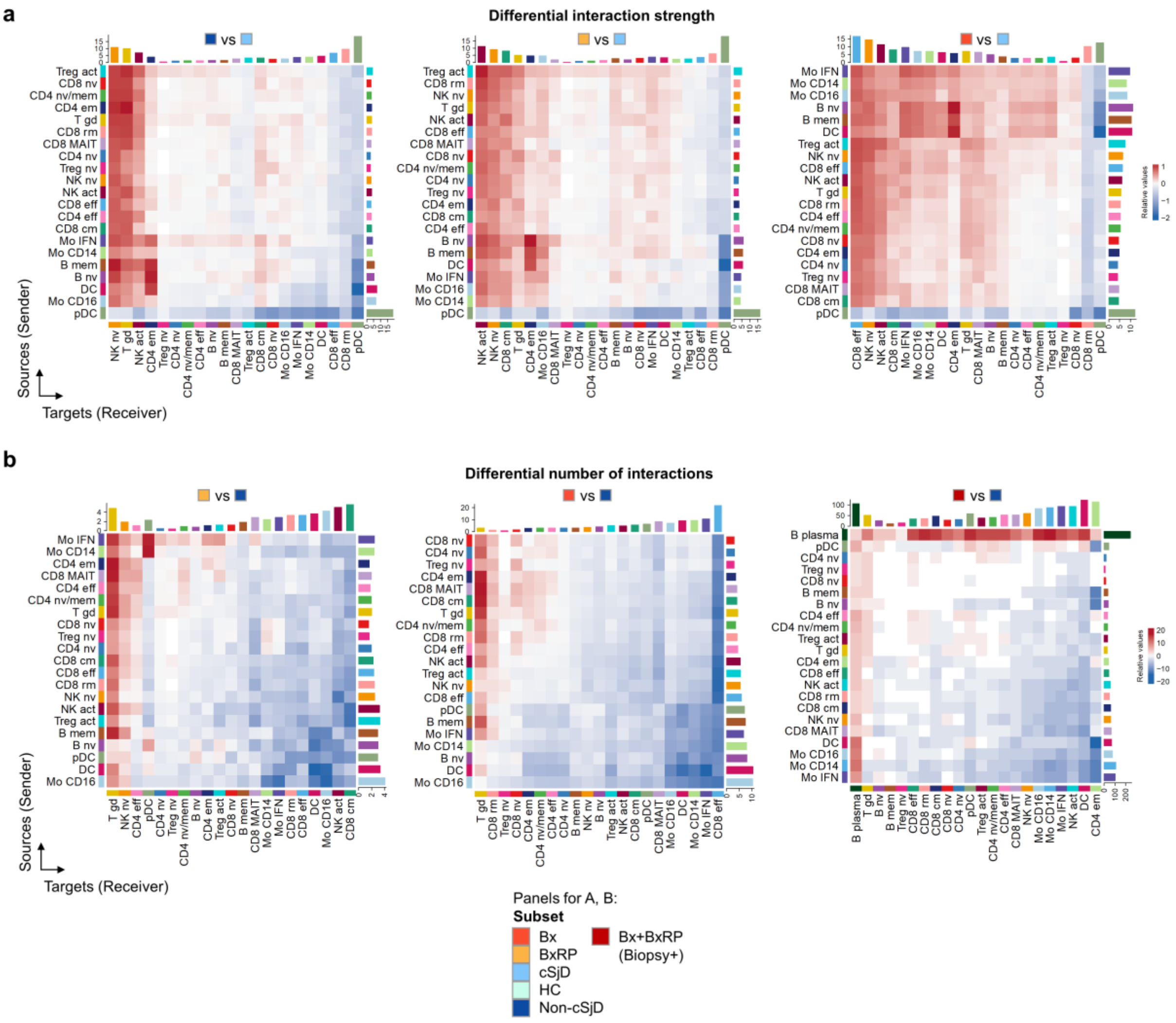

Supplementary Figure 11. CellChat-based identification of cell-to-cell interaction in cSjD and non-cSjD

a The overall pattern of differential interaction strength across the groups is shown. Differential interaction strength in cSjD when compared with other groups. Note that effector memory CD4^{+} T cells in cSjD are commonly upregulated in terms of signal-receiving from DC, naïve B cells, and memory B cells. **b** The overall pattern of differential interaction numbers across the groups is shown. Differential interaction numbers in either Bx, BxRP, or Biopsy^{+} (Bx and BxRP), compared to non-cSjD (biopsy-negative). Biopsy^{+} shows minimum cell-to-cell interactions when compared

with non-cSjD. Δ refers to differential changes. Relative values = the interaction strength/number from source to target in comparison group – the interaction strength/number from source to target in control group. Abbreviations: HC, healthy control; cSjD, childhood Sjögren's disease; BX, biopsy-positive non-cSjD without RP; BXRP, biopsy-positive non-cSjD with RP.

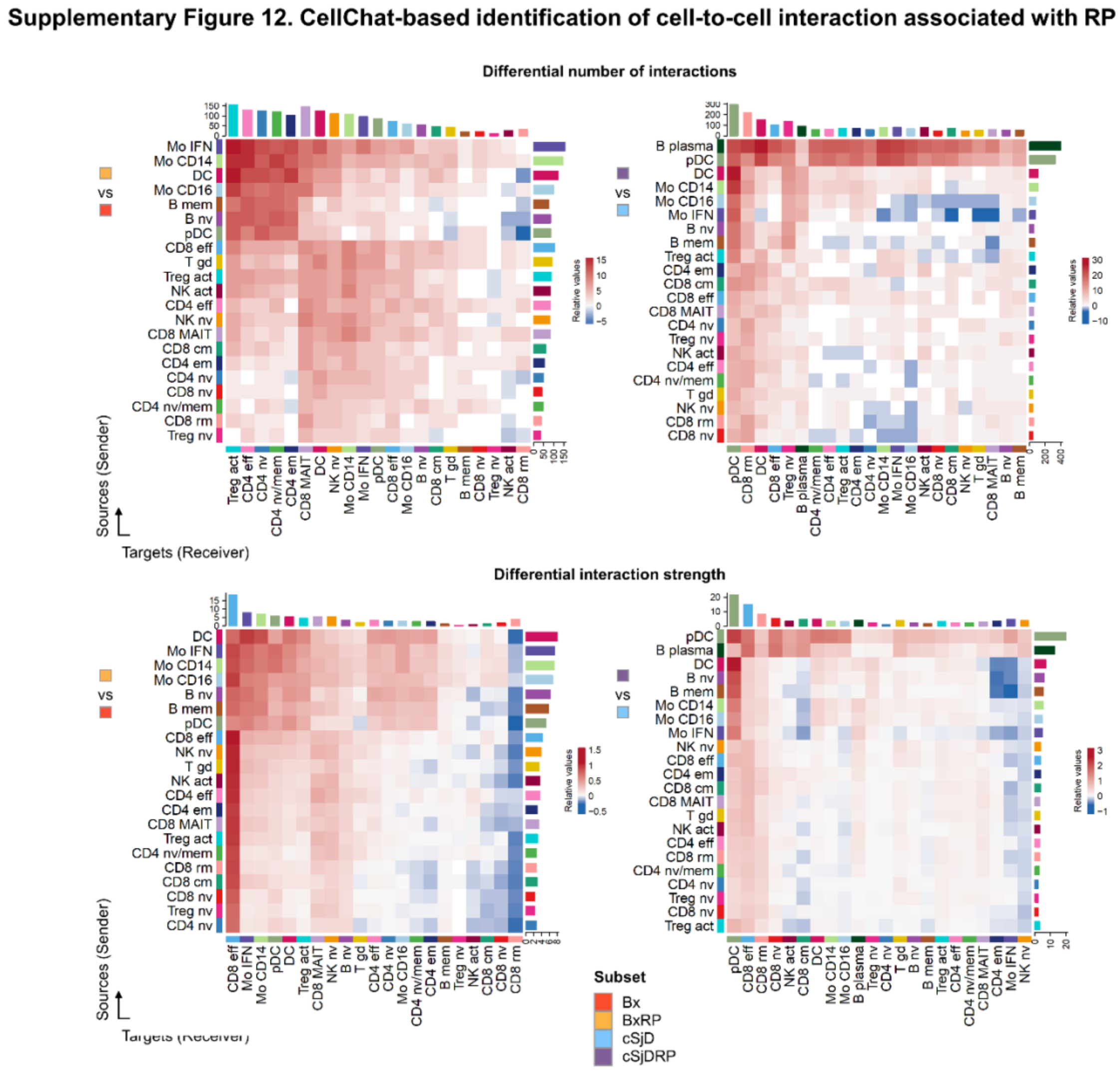

Supplementary Figure 12. CellChat-based identification of cell-to-cell interaction associated with RP

The overall pattern of differential interaction strength and numbers across the groups is shown. Relative values = the interaction strength/number from source to target in comparison group – the interaction strength/number from source to target in control group. Abbreviations: cSjD, childhood Sjögren’s disease; Bx, biopsy-positive non-cSjD without RP; BxRP, biopsy-positive non-cSjD with RP; cSjDRP, cSjD with RP.
