# Supplementary material for: Single-cell transcriptomics unveil profiles and interplay of immune subsets in rare autoimmune childhood Sjögren’s disease
Source: Commun Biol. 2024 Apr 19;7:481. doi: 10.1038/s42003-024-06124-6 (PMC11031574; doi:10.1038/s42003-024-06124-6)
Supplement: Supplementary file 3 — Description of Additional Supplementary Files [file 42003_2024_6124_MOESM3_ESM.pdf]

Description of Additional Supplementary Files

File name: Supplementary Data 1
Description: Doublet composition across sub-clustered immune subsets.

File name: Supplementary Data 2
Description: List of DEGs defining myeloid clusters in cSjD.

File name: Supplementary Data 3
Description: List of cSjD-related DEGs defining myeloid clusters.

File name: Supplementary Data 4
Description: List of cSjD-specific DEGs defining myeloid clusters.

File name: Supplementary Data 5
Description: List of biopsy-related DEGs defining myeloid clusters.

File name: Supplementary Data 6
Description: List of cluster biomarkers defining myeloid clusters in SjD.

File name: Supplementary Data 7
Description: List of DEGs defining cSjD and SjD Treg.

File name: Supplementary Data 8
Description: List of DEGs defining CD4+ clusters in cSjD.

File name: Supplementary Data 9
Description: List of cSjD-related DEGs defining CD4+ clusters.

File name: Supplementary Data 10
Description: List of cSjD-specific DEGs defining CD4+ clusters.

File name: Supplementary Data 11
Description: List of DEGs defining B clusters in cSjD.

File name: Supplementary Data 12
Description: List of cSjD-related DEGs defining B clusters in cSjD.

File name: Supplementary Data 13
Description: List of cSjD-specific DEGs defining B clusters in cSjD.

File name: Supplementary Data 14
Description: List of DEGs defining CD8+ T, γδ-T, and NK clusters in cSjD.

File name: Supplementary Data 15
Description: List of cSjD-related DEGs defining CD8+ T, γδ-T, and NK clusters in cSjD.

File name: Supplementary Data 16
Description: List of cSjD-specific DEGs defining CD8+ T, γδ-T, and NK clusters in cSjD.

File name: Supplementary Data 17
Description: List of biopsy-related DEGs defining CD8+ T, γδ-T, and NK clusters.

File name: Supplementary Data 18
Description: List of DEGs defining immune cell clusters related to recurrent parotitis.

File name: Supplementary Data 19
Description: Library and cell multiplexing statistics.

File name: Supplementary Data 20
Description: The source data behind the graphs in the figures.
