# Supplementary material for: Single-cell transcriptomics unveil profiles and interplay of immune subsets in rare autoimmune childhood Sjögren’s disease
Source: Commun Biol. 2024 Apr 19;7:481. doi: 10.1038/s42003-024-06124-6 (PMC11031574; doi:10.1038/s42003-024-06124-6)
Supplement: Supplementary file 5 — Reporting Summary [file 42003_2024_6124_MOESM5_ESM.pdf]

nature portfolio

Corresponding author(s): Seunghee Cha and Weizhou Zhang

Last updated by author(s): Feb 14, 2024

Reporting Summary

Nature Portfolio wishes to improve the reproducibility of the work that we publish. This form provides structure for consistency and transparency in reporting. For further information on Nature Portfolio policies, see our Editorial Policies and the Editorial Policy Checklist.

Statistics

For all statistical analyses, confirm that the following items are present in the figure legend, table legend, main text, or Methods section.

n/a	Confirmed	
☐	☒	The exact sample size (*n*) for each experimental group/condition, given as a discrete number and unit of measurement
☐	☒	A statement on whether measurements were taken from distinct samples or whether the same sample was measured repeatedly
☐	☒	The statistical test(s) used AND whether they are one- or two-sided<br>*Only common tests should be described solely by name; describe more complex techniques in the Methods section.*
☐	☒	A description of all covariates tested
☐	☒	A description of any assumptions or corrections, such as tests of normality and adjustment for multiple comparisons
☐	☒	A full description of the statistical parameters including central tendency (e.g. means) or other basic estimates (e.g. regression coefficient) AND variation (e.g. standard deviation) or associated estimates of uncertainty (e.g. confidence intervals)
☐	☒	For null hypothesis testing, the test statistic (e.g. *F*, *t*, *r*) with confidence intervals, effect sizes, degrees of freedom and *P* value noted<br>*Give P values as exact values whenever suitable.*
☒	☐	For Bayesian analysis, information on the choice of priors and Markov chain Monte Carlo settings
☒	☐	For hierarchical and complex designs, identification of the appropriate level for tests and full reporting of outcomes
☒	☐	Estimates of effect sizes (e.g. Cohen's *d*, Pearson's *r*), indicating how they were calculated

Our web collection on statistics for biologists contains articles on many of the points above.

Software and code

Policy information about availability of computer code

Data collection	All codes for single cell analysis will be available upon request.
Data analysis	Data analysis was performed by the Dr. Myung-Chul Kim. Software used: FlowJo (v 10.6.1); Graph Pad Prism 8.3.0; R(v 4.2.0); Cell Ranger package (v3.1.0); Seurat R package (v 4.2); SingleR Rpackage (v 1.8.1); Celldex Rpackage (v 1.6.0); scDblFinder Rpackage (v 1.4.0); escape Rpackage (v 1.6.0); DittoSeq Rpackage (v. 1.4.4); pheatmap Rpackage (v 1.0.12); Cell Ranger v 3.1.0 pipeline were used for all analysis. All analyses were further validated by Dr. Nicholas Borcherding who is an expert in bioinformatics and statistics.

For manuscripts utilizing custom algorithms or software that are central to the research but not yet described in published literature, software must be made available to editors and reviewers. We strongly encourage code deposition in a community repository (e.g. GitHub). See the Nature Portfolio guidelines for submitting code & software for further information.

Data

Policy information about availability of data

All manuscripts must include a data availability statement. This statement should provide the following information, where applicable:

- Accession codes, unique identifiers, or web links for publicly available datasets
- A description of any restrictions on data availability
- For clinical datasets or third party data, please ensure that the statement adheres to our policy

The raw and processed data from scRNA-seq in this study is available through the NIH dbGAP database with controlled access under the accession number

"phs003048.v1.p1". In addition, we included new analysis in our study from the publicly available datasets including: GSE148633, GSE206295, GSE168732, GSE148633, GSE216489, GSE211560, and NBDC hum0229.v.1.

Human research participants

Policy information about studies involving human research participants and Sex and Gender in Research.

Reporting on sex and gender	The terms sex and gender have not been collected in this study. We described participants male and female based on their sex.
Population characteristics	The diagnosis of childhood SjD (cSjD) was made according to the 2016 ACR/EULAR criteria for primary adult SjD and the age range of cSjD was from 10 to 17. Two cSjD patients exhibiting RP were included in the cSjD group. Those who did not fulfill the 2016 criteria were categorized as non-cSjD (ages ranging from 12 to 24). Interestingly, some non-cSjD patients presented positive lip biopsies without fulfilling the 2016 criteria, thus being classified as Bx (ages ranging from 8 to 18). Some of these biopsy-positive non-SjD patients presented RP, thus being categorized as BxRP (ages ranging from 7 to 19). Two subjects were older than 18 years old, more specifically 24 and 19, but included in this study as their disease onset was seven and eight years ago, respectively, thus still being seen by a pediatric rheumatologist. Young adolescents or young adults were recruited as health controls (HCs) (ages ranging from 18 to 31) since healthy children under 18 rarely visit rheumatology clinics in general.
Recruitment	Pediatric male and female participants who were referred from the Pediatric Rheumatology have been enrolled at the Center for Orphaned Autoimmune Disorders (COAD), UF Health/Shands, Gainesville, Florida.
Ethics oversight	All subjects provided written informed consent following the study protocol approved by the UF Institutional Review Board and the study was conducted in accordance with the Declaration of Helsinki.

Note that full information on the approval of the study protocol must also be provided in the manuscript.

Field-specific reporting

Please select the one below that is the best fit for your research. If you are not sure, read the appropriate sections before making your selection.

☒ Life sciences ☐ Behavioural & social sciences ☐ Ecological, evolutionary & environmental sciences

For a reference copy of the document with all sections, see nature.com/documents/nr-reporting-summary-flat.pdf

Life sciences study design

All studies must disclose on these points even when the disclosure is negative.

Sample size	Sample size is used depending on individual experiments, based on variation, statistical power and the nature of the experiments. For Treg suppression assay, two independent experiments for each child and adult group were performed. For single cell RNA seq data, we used Bonferroni adjusted P values. We normally use $P < 0.05$ to be considered statistically significant.
Data exclusions	All data are included except for flow data with no cells detected due to a technical error.
Replication	All experiments are labeled for biological replicates
Randomization	Four participant samples were allocated into one diagnostic group. These individuals were randomly selected from each diagnostic group. Five different diagnostic groups are formed based on the result of the lip biopsy (positive or negative) or the history of recurrent parotitis.
Blinding	It is not applicable to the present study on scRNA-seq.

Reporting for specific materials, systems and methods

We require information from authors about some types of materials, experimental systems and methods used in many studies. Here, indicate whether each material, system or method listed is relevant to your study. If you are not sure if a list item applies to your research, read the appropriate section before selecting a response.

Materials & experimental systems

n/a	Involved in the study
☐	☒ Antibodies
☒	☐ Eukaryotic cell lines
☒	☐ Palaeontology and archaeology
☒	☐ Animals and other organisms
☒	☐ Clinical data
☒	☐ Dual use research of concern

Methods

n/a	Involved in the study
☒	☐ ChIP-seq
☐	☒ Flow cytometry
☒	☐ MRI-based neuroimaging

Antibodies

Antibodies used

We used many antibodies, all from well-validated companies, such as flow antibodies that are mostly from BioLegend, eBioscience, BD bioscience. The list of antibodies used in this study is below (tab-delimited by Antibodies, Clone, Fluorescence, Source, and Cat #).
CD45 HI30 FITC BioLegend 304038
CD45 HI30 PE BioLegend 304039
CD45 HI30 BV421 BioLegend 304032
CD45 2D1 SPNIR685 BioLegend 368552
CD3 HIT3a FITC BioLegend 300306
CD3 SK7 PE-Fire 700 BioLegend 344864
CD4 OKT4 PerCP-Cy5.5 BioLegend 317428
CD4 RPA-T4 BV605 BioLegend 300556
CD4 SK3 PE-Cy5 BioLegend 344654
CD8 SK1 SB550 BioLegend 344760
CD25 M-A251 PE-Cy7 BioLegend 356108
CD127 A019D5 APC BioLegend 351316
CD127 A019D5 BV510 BioLegend 351334
CD152 BNI3 APC BioLegend 369612
FOXP3 259D Pacific Blue BioLegend 320216
FOXP3 206D Pacific Blue BioLegend 320116
CD11b M1/70 PE-Dazzle 594 BioLegend 101255
CD14 63D3 BV510 BioLegend 367124
CD14 HCD14 APC-Cy7 BioLegend 325620
CD16 3G8 BV785 BioLegend 302046
CD39 A1 PE-Cy7 BioLegend 328212
TIGIT MBSA43 EF710 Invitrogen 46-9500-42
Phospho STAT1 KIKSI0803 EF660 Invitrogen 50-9008-42
TotalSeq™-B0251 Hashtag 1 LNH-94 NA BioLegend 394631
TotalSeq™-B0252 Hashtag 2 LNH-94 NA BioLegend 394633
TotalSeq™-B0253 Hashtag 3 LNH-94 NA BioLegend 394635
TotalSeq™-B0254 Hashtag 4 LNH-94 NA BioLegend 394637
Mouse Anti-Human CD3 OKT3 NA BD Pharmingen 567107

Validation

Most of these antibodies are used hundreds of times in literature and validated from many publications. The company validates each batch before making them commercially available. The lab routinely validates new antibodies using established authentication methodologies.

Flow Cytometry

Plots

Confirm that:

☒ The axis labels state the marker and fluorochrome used (e.g. CD4-FITC).

☒ The axis scales are clearly visible. Include numbers along axes only for bottom left plot of group (a 'group' is an analysis of identical markers).

☒ All plots are contour plots with outliers or pseudocolor plots.

☒ A numerical value for number of cells or percentage (with statistics) is provided.

Methodology

Sample preparation	Peripheral blood mononuclear cells were isolated by density gradient using SepMate Tubes (StemCell Technologies).
Instrument	Aurora Cytek
Software	Flowjo
Cell population abundance	Live CD45+ peripheral mononuclear cells were sorted and confirmed by flow cytometric analysis.

Gating strategy	Examples were included for SSC/FSC gating. Negative/positive-gating was based on non-staining controls for well established antibodies and FMO for new antibodies used.

☒ Tick this box to confirm that a figure exemplifying the gating strategy is provided in the Supplementary Information.
